# Highly Targeted Detection of Priority Phytopathogen *Pectobacterium brasiliense*: From Obtaining Polyclonal Antibodies to Development and Approbation of Enzyme-Linked Immunoassay and Lateral Flow Immunoassay

**DOI:** 10.3390/microorganisms12122436

**Published:** 2024-11-27

**Authors:** Irina V. Safenkova, Pavel A. Galushka, Yuri A. Varitsev, Maria V. Kamionskaya, Natalia V. Drenova, Anna A. Vasilyeva, Anatoly V. Zherdev, Alexander I. Uskov, Boris B. Dzantiev

**Affiliations:** 1 A.N. Bach Institute of Biochemistry, Research Centre of Biotechnology of the Russian Academy of Sciences, 119071 Moscow, Russia; safenkova@inbi.ras.ru (I.V.S.); mari-kam@mail.ru (M.V.K.); zherdev@inbi.ras.ru (A.V.Z.); 2 Russian Potato Research Centre, 140051 Kraskovo, Moscow region, Russia; pavel_galushka@mail.ru (P.A.G.); varyuriy@yandex.ru (Y.A.V.); korenevo2005@mail.ru (A.I.U.); 3 All-Russian Plant Quarantine Centre, 140150 Bykovo, Moscow region, Russia; drenova@mail.ru; 4 Department of Plant Protection, Russian State Agrarian University—Moscow Timiryazev Agricultural Academy, 127434 Moscow, Russia; annadacyk@rgau-msha.ru

**Keywords:** ELISA, lateral flow immunoassay, phytopathogen, *Pectobacterium brasiliense*, polyclonal antibodies, potato soft rot

## Abstract

*Pectobacterium brasiliense* is a bacterial phytopathogen that causes soft and black rot and actively spreads worldwide. Our study is the first development of immunoassays for detecting *P. brasiliense*. We immunized rabbits and obtained serum with an extremely high titer (1:10^8^). Isolated polyclonal antibodies were tested by enzyme-linked immunosorbent assay (ELISA) using 18 closely related strains and 5 non-related bacterial pathogens. No cross-reactivity was found concerning the tested pathogens. The ELISA of *P. brasiliense* was developed in a double-antibody sandwich format with a detection limit of 1.5 × 10^4^ cells/mL. A lateral flow immunoassay (LFIA) for *P. brasiliense* was also developed in a double-antibody sandwich format with a detection limit of 1 × 10^5^ cells/mL. The results of *P. brasiliense* cells testing with LFIA in plant matrix showed a high correlation (R^2^ = 0.932) between concentrations of added and revealed cells. When testing potato seed material, ELISA and LFIA confirmed 75 and 66% of positive samples according to real-time PCR, respectively. For negative samples, ELISA showed 84% coincidence, and LFIA coincided with PCR for 89% of samples. Thus, the developed immunoassays can be used to evaluate plant material in poorly equipped conditions or under field testing.

## 1. Introduction

Bacterial blackleg, aerial stem rot, and tuber soft rot are devastating diseases of potatoes worldwide, primarily caused by *Pectobacterium* spp. and *Dickeya* spp. [1]. These bacterial pathogens secrete enzymes that destroy pectin and plant cell walls. The given processes lead to maceration and often wet and foul-smelling rot of plant organs [2]. *Pectobacterium brasiliense* is considered as one of the most virulent species, exhibiting high pathogenicity, and can cause blackleg, aerial stem rot, and tuber soft rot [3,4]. *P. brasiliense* was originally identified in Brazil and was initially named *Erwinia carotovora* subsp. *brasiliensis* [5]. Based on molecular and phylogenetic studies, the taxon identified as *P. carotovorum* subsp. *brasiliense* has been elevated to the species level of *P. brasiliense* [6,7]. However, the pathogen has rapidly become a global problem, with cases reported in various countries in North America, Europe, Africa, and East Asia [8,9,10,11,12,13]. Moreover, the pathogenicity of *P. brasiliense* extends beyond the disease of potato (*Solanum tuberosum* L.). At least 19 affected agricultural crops of high practical importance are known to date [4,14]. Bacterial species from the *Pectobacteriaceae* family are among the most significant threats to both seed and ware potato production, causing substantial economic losses. For instance, the total estimated loss for the European potato sector each year is approximately 46 million euros [15]. *P. brasiliense* has emerged as one of the dominant species in this family, indicating that a significant portion of these economic losses can be attributed to *P. brasiliense*.

The increasing prevalence of *P. brasiliense* worldwide, coupled with the significant damage caused by this pathogen, necessitates effective tools for detecting *P. brasiliense* in plant material. Primary screening testing methods and methods for precise identification and quantitative characterization are used to assess the contamination of plant material with bacterial infections [16,17,18,19,20]. For precise laboratory identification, molecular diagnostic methods based on recognition and subsequent amplification of specific bacterial DNA fragments are usually used. This group includes both conventional and TaqMan-based PCR techniques that are developed for detecting *P. brasiliense* [5,21,22,23,24]. High sensitivity is the main advantage of PCR methods, but their limitation is strict requirements for laboratory equipment and analysis conditions. In particular, PCR diagnostics require a thermal cycler, equipping the laboratory with devices that ensure cleanliness, and specially equipped room; additional costs for qualified specialists should also be considered. As for screening control methods, their place is mainly occupied by immunochemical methods based on the specific binding of antigenic determinants on the surface of bacteria with antibodies. Enzyme-linked immunosorbent assay (ELISA) and lateral flow immunoassay (LFIA) are the most widespread immunochemical methods that are capable of rapid screening directly at the point of need (phytosanitary control sites, field monitoring, seed testing) or in laboratories with poor technical equipment. Immunochemical methods for detecting *Pectobacterium* spp. and *Dickeya* spp. have been proposed [25,26]. However, to date, none of the immunochemical methods have been developed for detection of *P. brasiliense* and its differentiation from closely related species. This is probably partly due to the recent allocation of *P. brasiliense* as a separate species, and partly due to the difficulty of obtaining highly selective antibodies to one of the representatives of a large group of closely related bacteria. The goal of the presented study was to obtain polyclonal antibodies specific to *P. brasiliense* and develop immunoanalytical systems based on them such as ELISA, and LFIA. The potential of the developed test systems was demonstrated in testing potato tuber samples for the presence of *P. brasiliense*.

## 2. Materials and Methods

### 2.1. Materials

This study used sodium tetrachloroaurate; sodium citrate; sodium azide; bovine serum albumin (BSA), horseradish peroxidase, protein A-Sepharose CL-4B, and incomplete Freund’s adjuvant (Sigma-Aldrich, St. Louis, MO, USA); Triton-X-100, and Tween-20, (DIA-M, Moscow, Russia); glycerol (Reakhim, Moscow, Russia); a substrate mixture for peroxidase activity based on 3,3′,5,5′-tetramethylbenzidine (TMB) and hydrogen peroxide (Immunotech, Moscow, Russia); horseradish peroxidase conjugates with goat antibodies against rabbit immunoglobulins G (Imtek, Moscow, Russia); tris(hydroxymethyl)aminomethane (Loba Chemie, Mumbai, Maharashtra, India). All the salts and organic compounds used were of analytical-grade purity. A Simplicity Milli-Q^®^ system from Millipore (Burlington, MA, USA) was used to obtain ultrapure water for buffers and reagent solutions.

For the fabrication of lateral flow test strips, CN-95 nitrocellulose membranes (Sartorius Stedim Biotech, Göttingen, Germany), AP045 final absorbent membrane, PT-R5 glass fiber membranes for conjugate, GFB-R4 absorbent membranes for samples and L-P25 plastic backing (Advanced Microdevices, Ambala Cantt, Haryana, India) were used. For ELISA, 96-well transparent polystyrene microplates (Costar 9018, Corning Costar, Glendale, AZ, USA) were used.

### 2.2. Bacterial Strains

For rabbit immunization and all specific testing, *Pectobacterium brasiliense* F126 [11] was used from the collection at the Laboratory of Molecular Bioengineering of the Shemyakin-Ovchinnikov Institute of Bioorganic Chemistry, Russian Academy of Sciences, as described earlier [27]. For specificity testing, we used bacterium strains obtained from the collection of phytopathogens of the Leibniz Institute DSMZ–German Collection of Microorganisms and Cell Cultures (DSM), the National Collection of Plant Pathogenic Bacteria (NCPPB) the collection of phytopathogens of the All-Russian Research Institute of Phytopathology (VNIIF), the bacterial collection of the All-Russian Plant Quarantine Center (VNIIKR), the French Collection for Plant-associated Bacteria (CFBP): *P. atrosepticum* (DSMZ 18077, VNIIF 5128, VNIIKR 0143), *P. wasabie* (DSM 18074), *P. betavasculorum* (DSM 18076), *P. carotovorum* (VNIIF 3391, VNIIF 3398, VNIIP C966, DSM 30168), *P. odoriferum* (DSM 22566), *Clavibacter sepedonicus* (VNIIKR 0435, VNIIKR 0668), *C. michiganensis* (VNIIKR 0496), *Dickeya solani* (DSM 28711), *D. dadantii* subsp. *dadantii* (DSM 18020), *D. paradisiaca* (DSM 18069), *D. fangzhongdai* (DSM 101947), *D. dadantii* subsp. *dieffenbachiae* (DSM 18012), *D. dianthicola* (CFBP 3705, VNIIKR 0144), *D. zeae* (DSM 18068), *Ralstonia solanacearum* (race 3, bv.2, NCPPB 2316), *Pseudomonas fluorescence* (VNIIKR 0213).

### 2.3. Preparation of Potato Tuber Extracts

To analyze potato tubers, the extracts were prepared using the following protocol: cones of vascular tissue 3–5 mm in diameter were cut from each batch of potato tubers. The samples were placed in a flask and grounded, sterile 50 mM phosphate buffer, pH 7.0, was added, and large particles were removed by filtration. The resulting extract was then centrifuged at 10,000× *g* for 10 min, 4 °C; after removing the supernatant, the sediment was resuspended in the 50 mM phosphate buffer, pH 7.0 with 0.05% Triton X-100 and used for analysis.

### 2.4. Obtaining Serum and Isolating Polyclonal Antibodies

To obtain antisera, Chinchilla rabbits were immunized with a 48 h culture of *P. brasiliense* in an amount of 10^9^ cells per injection. Incomplete Freund’s adjuvant was used for immunization at weekly intervals, the 1st and 6th injections were subcutaneous, and the 2nd through 5th injections were intramuscular. Blood was collected on the tenth day after the last injection according to [26]. Immunoglobulins G (IgG) were isolated from the serum by precipitation with a saturated ammonium sulfate solution, followed by affinity chromatography on a column with protein A-Sepharose CL-4B. The concentration of isolated antibodies was assessed spectrophotometrically, and specificity was characterized using the enzyme-linked immunosorbent assay described in the Section 2.7. The isolated antibody preparations were stored in 50 mM phosphate buffer with the addition of 0.05% sodium azide at +4 °C.

### 2.5. Preparation of Conjugates of Antibodies with Horseradish Peroxidase

Polyclonal antibodies were conjugated with horseradish peroxidase. To obtain conjugates of isolated IgG with horseradish peroxidase, the periodate synthesis method was used according to [28]. The conjugate was characterized spectrophotometrically in the wavelength range from 240 to 500 nm and the absorbance was determined at 403 nm (the heme-mediated peroxidase absorption peak) and 280 nm (the IgG and peroxidase absorption peak). The specificity of the conjugates was characterized by ELISA as described in the Section 2.7, using *P. brasiliense* as a positive control, *D. solani*, *C. sepedonicus*, *E. amylovora*, *P. carotovorum, D. dianthicola*, *R. solanacearum*, *P. atrosepticum* as negative controls. The antibody-peroxidase conjugate was stored in 50 mM phosphate buffer with 50% glycerol at –20 °C.

### 2.6. Preparation of Conjugates of Antibodies with Gold Nanoparticles

Gold nanoparticles (GNPs) with a diameter of 25 nm were synthesized by the citrate reduction method [29], with slight modification. One milliliter of 1% HAuCl_4_ was added to 97.5 mL of deionized water. The mixture was continuously stirred and heated to the boiling point; then, 1.5 mL of 1% sodium citrate was added. The GNP solution was continuously boiled for another 30 min. After cooling, GNPs were conjugated with polyclonal antibodies specific to *P. brasiliense* using physical adsorption according to the protocol described by Byzova et al. [30]. The antibody conjugate with GNPs was stored in 10 mM Tris buffer, pH 7.4, containing 0.25% BSA, 0.25% Tween 20, 1% sucrose, and 0.05% sodium azide at +4 °C.

The GNPs were characterized by transmission electron microscopy (TEM), dynamic laser light scattering (DLS), and spectrophotometry. The conjugate was characterized using DLS and spectrophotometry. For the characterization of GNPs by TEM, a JEM CX-100 transmission electron microscope (Jeol, Tokyo, Japan) at 80 kW and a magnification of 33,000–100,000 was used. The resulting micrographs of nanoparticles were scanned, and the average diameter of nanoparticles was determined using the Image Tool program (University of Texas Health Science Center at San Antonio, TX, USA). The spectra of the GNPs and their conjugate were recorded using a Libra S80 spectrophotometer (Biochrom, UK). Hydrodynamic diameters of GNPs and their conjugate were measured using a Zetasizer Nano ZSP (Malvern Panalytical, Malvern, UK).

### 2.7. Characterization of Sera and Isolated Antibodies by Enzyme-Linked Immunoassay

*P. brasiliense* cells (100 μL of 1 × 10^8^ cells/mL in 50 mM phosphate-buffered saline, pH 7.4, 100 mM NaCl [PBS]) were adsorbed in the microplate wells for 2 h at 37 °C. Unbound reagents were removed by washing the wells three times with PBS with 0.05% Triton X-100 (PBST) using Thermo Electron WellWash 4 MK2 (ThermoScientific, Waltham, MA, USA). After washing, sera (diluted from 10^4^ to 10^9^ in PBST) or isolated IgG (from 0.1 to 400 ng/mL in PBST) were added to the wells and incubated for 1 h at 37 °C. The microplate was washed three times with PBST, after which peroxidase conjugate with goat antibodies against rabbit immunoglobulins G (commercial preparation diluted 1:5000 in PBST) was added and incubated for 1 h at 37 °C. After washing, 100 μL of the substrate mixture containing TMB and H_2_O_2_ were added to the wells of the microplate and incubated for 15 min at room temperature. The enzymatic reaction was stopped by adding 50 μL of 1 M sulfuric acid. Optical density was measured at a wavelength of 450 nm (OD_450_) using a Multimode Plate Reader—Enspire microplate spectrophotometer (PerkinElmer, UK). The obtained data were processed using the OriginPro 2021 software (OriginLab, Northampton, MA, USA). The serum titer was considered to be the dilution value equal to the sum of the mean OD_450_ value and three standard deviations for the serum reaction with the negative control.

### 2.8. Double-Antibody Sandwich Enzyme-Linked Immunosorbent Assay

Polyclonal antibodies specific to *P. brasiliense* (100 μL of 2 μg/mL in PBS) were adsorbed into the microplate wells for 2 h at 37 °C or at 4 °C overnight. The unbound reagents were removed by washing the wells three times with PBST. Then, 100 μL of sample were added to the wells and incubated for 1 h at +37 °C. Then, washing was performed again, and 100 μL of polyclonal antibodies (1 μg/mL)—peroxidase conjugate in PBST were added and incubated at 37 °C for 1 h. After washing, the addition of TMB substrate mixture and recording of results were carried out as described in the Section 2.7.

For calibration curve and concentration dependences of cross-reactants, the samples were bacterial cells (*P. brasiliense, P. carotovorum, P. odoriferum, P. atrosepticum*) in PBST (bacterial concentration—from 0 to 1 × 10^7^ cells/mL).

For the selectivity test, the samples were 24 bacteria (10^8^ cells/mL) that are listed in the Section 2.2 and also potato tuber extracts.

For testing natural tuber material, the samples were potato tuber extracts obtained from seed potatoes according to the Section 2.3. Bacterial cells of *P. brasiliense* at a concentration of 10^7^ cells/mL, added to a tuber extract of a healthy plant, were used as a positive control, and a tuber extract of a healthy plant was used as a negative control.

### 2.9. Preparation of Lateral Flow Test Strips

Lateral flow test strips were assembled according to the scheme and protocol described in [31]. Polyclonal antibodies specific to *P. brasiliense* (1 mg/mL in PBS with 5% glycerol and 0.03% NaN_3_) were applied to the test zone, and protein A (0.5 mg/mL in PBS with 5% glycerol and 0.03% NaN_3_) was applied to the control zone at a rate of 0.15 μL per mm using an IsoFlow dispenser (Imagene Technology, St. Lebanon, NH, USA). The GNP-antibody conjugate (OD_520_ = 3.5) was applied to a glass fiber membrane (5 mm wide) at a rate of 3.2 μL per mm. The membranes were dried at 37 °C for at least 6 h, after which a multimembrane composite was collected, consisting of an absorbent membrane for the sample, a membrane with the conjugate, a nitrocellulose membrane, and a final absorbent membrane. To obtain lateral flow test strips (3 mm wide), the multimembrane composite was cut using an automatic guillotine ZQ2002 (Shanghai Kinbio Tech, Shanghai, China). The test strips were packaged in laminated aluminum foil pouches containing silica gel and stored at room temperature.

### 2.10. Lateral Flow Immunoassay

The lateral flow test strip was immersed directly into the analyzed sample (buffer or extract) and the result was visually assessed after 15 min. The appearance of two-colored lines indicated the presence of pathogen in the sample, only one line in the control zone indicated the absence of bacteria in the sample or its presence, but at a concentration below the detection limit of the test strips.

The test strips were scanned using a Canon 9000F Mark II scanner (Canon, Tokyo, Japan). The obtained digital data were processed using TotalLab TL120 (Nonlinear Dynamics, Newcastle, UK) to obtain the values of color intensities in the test zones of test strips.

For calibration curves, the samples were bacterial cells (*P. brasiliense*) in PBST and potato tuber extract from healthy potato (bacterial concentration—from 0 to 1 × 10^8^ cells/mL).

For the selectivity test, the samples were bacterial cells (10^8^ cells/mL of *D. solani*, *C. sepedonicus*, *E. amylovora*, *P. carotovorum, D. dianthicola*, *R. solanacearum*, *P. atrosepticum*) in potato tuber extracts from healthy potato.

For testing natural tuber material, the samples were potato tuber extracts obtained from seed potatoes according to the Section 2.3. Bacterial cells of *P. brasiliense* at a concentration of 10^7^ cells/mL, added to a tuber extract of a healthy plant, were used as a positive control, and a tuber extract of a healthy plant was used as a negative control.

### 2.11. Real-Time PCR Analysis

Bacterial DNA was isolated from tuber extracts using a commercial kit (PH-520, Syntol, Moscow, Russia). A commercial qPCR kit (Pecto Dif-PB, Syntol, Moscow, Russia) intended for *P. brasiliense* detection was used according to the manufacturer’s protocol with cycle threshold (Ct) equaled to 40. The measurements of fluorescence at 533 nm of excitation and 572 nm of emission were carried out with the Roche Light Cycler 96 (Roche, Rotkreuz, Switzerland).

### 2.12. Statistical Data Analysis

All ELISA experiments were performed at least three times, and all LFIA experiments were performed at least two times. Mean values, standard deviations, and approximations with 4-parameter sigmoid function for the concentration dependences were calculated by OriginPro 2021 (OriginLab, Northampton, MA, USA). The three-sigma method was used to determine the detection limit.

T-tests were performed by GraphPad Prism version 8.0 (GraphPad Software, Boston, MA, USA) for two cases: (1) ELISA of *P. brasiliense* and closely related species of *Pectobacterium* at high concentrations, (2) LFIA of samples with different amounts of *P. brasiliense* cells in buffer and tuber extract from healthy potatoes.

McNemar’s test was used to assess the level of agreement between ELISA and PCR, LFIA and PCR. The GraphPad Prism software was applied for this purpose with the following interpretation of the results: If the compared assays demonstrate a statistically significant difference in McNemar’s test, this reflects a serious disagreement between the assays.

Additionally, to compare assay pairs, we calculated the true positive rate (TPR), expressed as TPR = TP/(TP + FN), where TP is the number of true positive instances, and FN is the number of false negative instances. The false positive rate (FPR) was calculated as FP/(FP+TN), where FP is the number of false positives and TN is the number of true negatives.

## 3. Results and Discussion

### 3.1. Characteristics of the Obtained Sera and Isolated IgG

**Figure 1:**
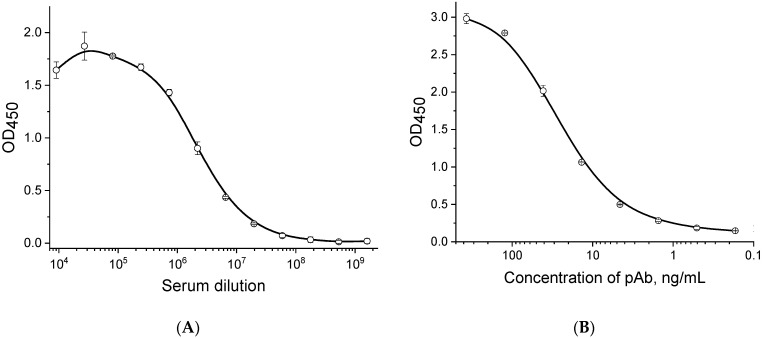
Characteristics of serum (**A**) and isolated IgG fraction (**B**) specific to *P. brasiliense* using the indirect ELISA method with sorption of *P. brasiliense* bacteria in microplate wells at a concentration of 1 × 10^8^ cells/mL. Points at the curves are the average values of three replicates; error bars represent standard deviations.

As a result of the immunization of rabbits, a serum was obtained from blood taken on the 10th day after the last injection of bacterial cells. The specificity and titer of the serum were determined by indirect ELISA. The results are shown in Figure 1A. Figure 1A demonstrates that the titer of the serum was equaled to 1:10^8^. The non-specific titer of serum against a closely related pathogen, the bacterium *P. carotovorum*, which also causes blackleg symptoms, was significantly worse and equal to 1:10^5^.

The antibodies (fraction of immunoglobulins G) isolated from the serum were confirmed to have high antigen-binding activity against *P. brasiliense* using the indirect ELISA method; the results are shown in Figure 1B. The antibodies starting from a concentration of 0.5 ng/mL and above recognized *P. brasiliense* cells adsorbed in the microplate wells. The obtained concentration dependence of the antibodies (see Figure 1B) indicates a significant amount of the high-affinity IgG fraction in the preparation of the isolated antibodies.

Based on the results obtained, it can be expected that the isolated antibodies are promising for the development of ELISA and LFIA systems. The double-antibody sandwich format of immunoassay is the most effective for ELISA and LFIA of bacteria that are polyvalent antigens [32]. Therefore, we used the isolated antibodies in two variants: (1) as capture antibodies, adsorbing them in the microplate wells for ELISA and in the test zone of lateral flow test strips, and (2) as detecting labeled antibodies that were conjugated with peroxidase for ELISA and with gold nanoparticles (GNPs) for LFIA. In both techniques, a triple [capture antibodies]—[bacteria]—[conjugate of antibody with label] complex is formed in the presence of bacteria.

### 3.2. Development of an ELISA Test System

A conjugate of the isolated IgG with peroxidase was synthesized with a minimal antibody:peroxidase ratio (<1:1 according to spectrophotometry data, see Supporting Information, Figure S1). This maintained the high sensitivity of the conjugate to *P. brasiliense* and the absence of cross-reactivity to other pathogens. At a conjugate concentration of 1–5 μg/mL, an acceptable high ratio of specific/non-specific signals was observed (Supporting Information, Figure S2).

**Figure 2:**
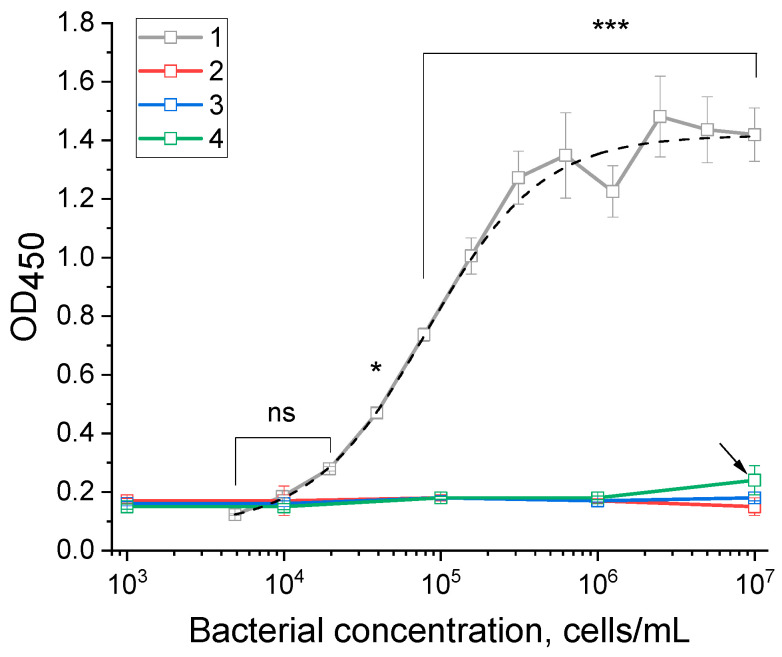
Concentration dependences for *P. brasiliense* (1), *P. carotovorum* (2), *P. odoriferum* (3), and *P. atrosepticum* (4), obtained in a double-antibody sandwich ELISA. Points at the curves are the average values of four replicates; error bars represent standard deviations. The dotted curve approximates the concentration dependence for *P. brasiliense*, R^2^ = 0.997. Difference between detection of different concentrations of *P. brasiliense* and detection of closely related species of *Pectobacterium* at high concentration (the value is indicated by the arrow) according to *t*-test: ns—*p*-value > 0.05, *—*p*-value > 0.01, ***—*p*-value < 0.001.

In the ELISA test system, the optimal concentrations of reagents providing the maximum signal-to-noise ratio corresponded to 2 μg/mL of capture antibodies immobilized in the wells of the microplate, 1 μg/mL of antibody-peroxidase conjugate. Under the selected conditions, ELISA was carried out for four *Pectobacterium* species (*P. brasiliense*, *P. carotovorum*, *P. odoriferum*, *P. atrosepticum*) in the concentration range from 10^7^ to 10^3^ cells/mL. The reliable response was observed only for *P. brasiliense* cells, for the other three *Pectobacterium* species the signal corresponded to the background level for all tested concentrations (Figure 2). The curve was approximated with 4-parameter sigmoid function with R^2^ equal to 0.997 [y = A2 + (A1 − A2)/(1 + (x/x0)^p^), where A1 = 0.08; A2 = 1.42; x0 = 8.1 × 10^4^; *p* = 1.19]. The detection limit (3σ) for *P. brasiliense* was 1.5 × 10^4^ cells/mL.

To confirm the high selectivity of the obtained antibodies, the ELISA technique was applied to an extended panel of bacterial collection, including 18 closely related strains of *Pectobacterium* and *Dickeya* and 5 non-related pathogens from *Ralstonia*, *Clavibacter*, and *Pseudomonas* genus. The closely related strains also belong to the *Pectobacteriaceae* family and cause similar symptoms (blackleg and tuber soft rot) [1]. Identification of *P. brasiliense* among them is crucial for accurately determining the cause of the disease. Bacteria related to *Ralstonia* and *Clavibacter* are also among the most common potato pathogens, like *P. brasiliense*. *Pseudomonas fluorescens* is a plant growth-promoting rhizobacterium that has potential as a bio-pesticide for enhancing biological control of various agricultural and horticultural diseases. The likelihood of cross-reactivity with these bacteria is low; however, they were tested starting from a high concentration of 10^8^ cells/mL. As a result, ELISA showed a strong positive result only for *P. brasiliense* (Table 1). For all other bacteria, including strains belonging to the genus of *Pectobacterium*, *Dickeya*, the signal values did not exceed the background (0.25) (see Table 1). Therefore, we assume that ELISA based on the obtained antibodies does not have cross-reactivity with closely related bacteria. For healthy plant material (potato tuber extract), no cross-reactive signals were observed. When *P. brasiliense* cells were added to the potato tuber extract, a strong positive signal was detected, which was not inferior to the signal when detecting *P. brasiliense* in the buffer. Thus, for ELISA based on polyclonal antibodies, a unique highly targeted specificity was demonstrated for *P. brasiliense* cells. The obtained high specificity is not typical for polyclonal antibodies. As a rule, polyclonal antibodies are not highly specific, since previously obtained antibodies and test systems had broad specificity in several species of *Dickeya* (*D. solani*, *D. dianthicola*, *D. chrysanthemi*, *D. dadantii*, *D. paradisiaca*) and *Pectobacterium* [25,26].

### 3.3. Development of an LFIA Test System

In developing the LFIA, we synthesized spherical GNPs, which are the detecting labels in the analysis. According to transmission electron microscopy (TEM), the diameter of the nanoparticles was 24.4 ± 3.9 nm, with an ellipticity degree of 1.2 ± 0.2 (the micrograph of GNPs and the diameter distribution histogram are shown in Supporting Information, Figure S3). The absorption spectrum of GNPs showed a maximum at a wavelength of 523 nm (Supporting Information, Figure S4), which also confirms the size of the synthesized nanoparticles [33]. The homogeneity of GNPs and the absence of aggregates in the solution were confirmed by dynamic laser light scattering (DLS)—the main peak corresponded to particles with a hydrodynamic diameter of 34.0 nm (Supporting Information, Figure S4).

Based on the synthesized GNPs, conjugates with polyclonal antibodies were obtained by physical adsorption of antibodies on the surface of the nanoparticles. Spectrophotometric characterization of the conjugates showed a peak shift to the long-wave region (the absorption maximum is 528 nm, Supporting Information, Figure S4), which indicates successful immobilization of antibodies on the surface of nanoparticles [34]. The hydrodynamic size of the conjugates also showed an increase in size relative to the nanoparticle size (the average hydrodynamic diameter was 78.7 nm, Supporting Information, Figure S4).

**Figure 3:**
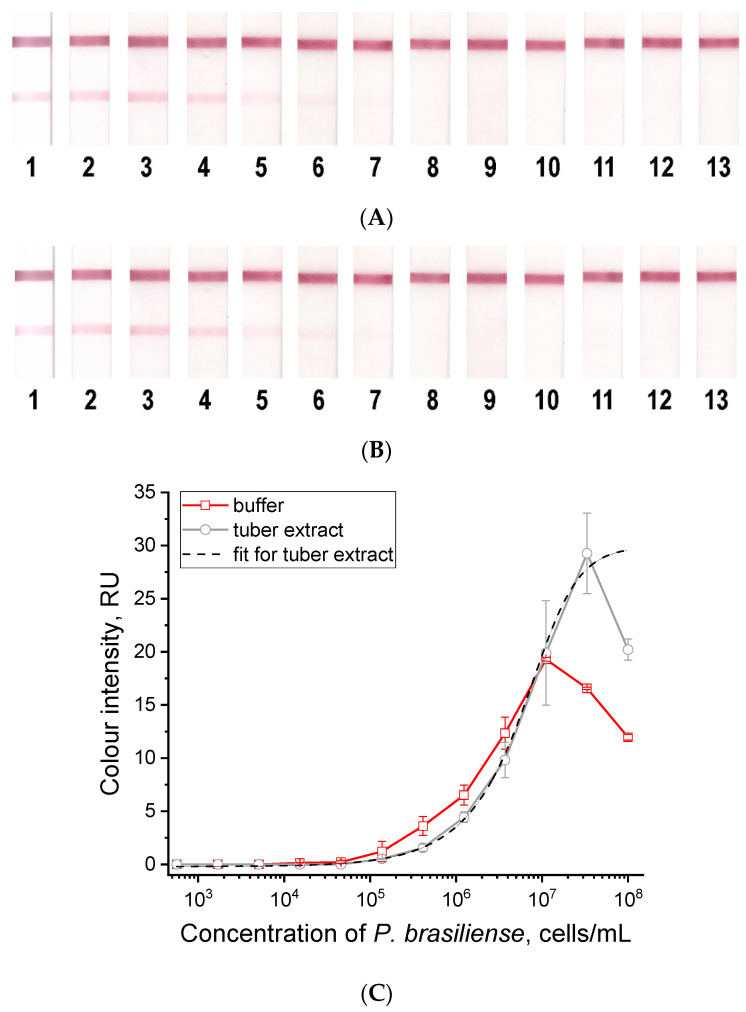
Test strips after analysis of samples containing different numbers of *P. brasiliense* cells (1—1 × 10^8^, 2—3.3 × 10^7^, 3—1.1 × 10^7^, 4—3.7 × 10^6^, 5—1.2 × 10^6^, 6—4.1 × 10^5^, 7—1.4 × 10^5^, 8—4.6 × 10^4^, 9—1.5 × 10^4^ cells/mL, 10—5.1 × 10^3^, 11—1.7 × 10^3^, 12—5.7 × 10^2^ cells/mL, and 13—negative control) in buffer (**A**) and tuber extract from healthy potato (**B**) and the corresponding dependences of the intensity of the recorded signals of the test zones on the concentration of *P. brasiliense* (**C**). Points at the curves are the average values of two replicates; error bars represent standard deviations. The dotted curve approximates the concentration dependence for *P. brasiliense* in tuber extract, R^2^ = 0.999. The reduction in color intensity at high concentrations is due to a hook effect specific to sandwich immunoassay formats, indicating that some bacteria–conjugate complexes cannot bind in the test zone occupied by bacteria.

The resulting series of lateral flow test strips were tested in buffer and healthy potato tuber extracts with the addition of *P. brasiliense* at concentrations from 10^8^ to 10^2^ cells/mL. In both cases, concentration dependences were obtained that had a similar appearance (Figure 3). Moreover, the *t*-test confirmed (*p*-value = 0.6855) the convergence of the results obtained with LFIA in buffer and plant extracts. For both curves, a decrease in signal was characteristic at high concentrations (above 1.1 × 10^7^ cells/mL for the buffer and above 3.3 × 10^7^ cells/mL for the tuber extract). This hook effect manifested in sandwich immunoassay formats indicates that at high antigen concentrations, some of the bacteria–conjugate complexes are unable to bind in the test zone occupied by bacteria. The curve for tuber extract at the range of 3 × 10^7^ to 10^2^ cells/mL was approximated with 4-parameter sigmoid function with R^2^ equal to 0.999 [y = A2 + (A1 − A2)/(1 + (x/x0)^p^), where A1 = 0.0; A2 = 31.5; x0 = 6.5 × 10^6^; *p* = 1.17]. For quantitative evaluation, we recommend using concentrations in the range from 1 × 10^5^ to 5 × 10^7^ cells/mL. Due to the observed hook effect, it is advisable to perform a 10-fold dilution if a quantitative analysis is necessary. This means analyzing both the original sample and a sample diluted 10 times with the working buffer. This approach will ensure that the concentration of the detected bacteria in the sample is accurately determined. If only a qualitative analysis is needed, a 10-fold dilution of the sample is not required. The hook effect does not result in a zero signal, even at very high concentrations of bacteria in the sample (see Figure 3). For the LFIA test system, the detection limit (3σ) for *P. brasiliense* was 1 × 10^5^ cells/mL. The obtained detection limit for LFIA is approximately 7 times higher than for ELISA. At the same time, the obvious advantage of LFIA over ELISA is the shorter analysis time, which was 15 min compared to 2.5 h, and the possibility of out-of-laboratory testing.

When testing healthy plant material (tubers), the LFIA test systems showed no background signals (see Figure 3). The cross-reactive signals were assessed by testing the main plant pathogens (*Dickeya solani*, *Clavibacter sepedonicus*, *Erwinia amylovora*, *Pectobacterium carotovorum*, *Dickeya dianthicola*, *Ralstonia solanacearum*, *Pectobacterium atrosepticum*) at high concentrations (10^8^ cells/mL). For all bacteria, zero signal values were obtained in the LFIA (Supporting Information, Table S1).

**Figure 4:**
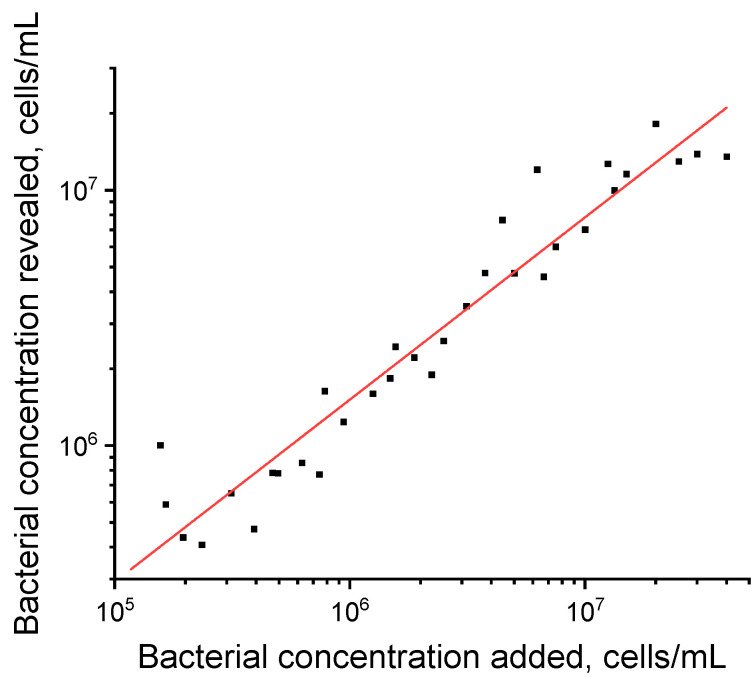
The correlation of the concentrations of *P. brasiliense* added in a tuber extract and revealed by LFIA are shown on the X and Y axes, respectively. R^2^ = 0.932.

To evaluate the possibilities of quantitative interpretation of LFIA results, we obtained 34 samples by adding *P. brasiliense* to the tuber extract of a healthy plant. As recommended above, concentrations in the range from 1 × 10^5^ to 5 × 10^7^ cells/mL were used for the experiment (see Figure 3C). The obtained values of *P. brasiliense* concentrations had a good linear correlation (R^2^ = 0.932) with the added values; data are presented in Figure 4. These results indicate that LFIA can be used to quantitatively evaluate *P. brasiliense* content in samples.

### 3.4. Testing Natural Tuber Material with the Developed ELISA and LFIA Test Systems

Seed potato lots obtained from different regions of Kyrgyzstan and Russia were tested for the presence or absence of *P. brasiliense* using the developed ELISA, LFIA, and commercial real-time PCR system. Out of 43 samples (each sample is total for a lot of 100 tubers), real-time PCR detected 24 infected samples (fluorescence data are presented in Supporting Information, Figure S5), ELISA—21, and LFIA—18 (the results are presented in Table 2). However, ELISA and LFIA confirmed positive samples determined by real-time PCR in 75 and 66% of cases, respectively. For negative samples, ELISA showed 84% coincidence, and LFIA coincided with real-time PCR for 89% of samples. Thus, despite the lack of cross-reactivity with the main closely related pathogens, ELISA and LFIA detected 2 and 1 samples as positive, while commercial real-time PCR did not detect *P. brasiliense* in them. To estimate the sensitivity of the assays, we calculated TPR, which was 0.75 for ELISA and 0.66 for LFIA. For specificity, FPR was found to be 0.16 for ELISA and 0.11 for LFIA. McNemar’s test results showed no significant differences between ELISA and PCR (χ^2^ = 0.444, df = 1, *p* = 0.5050), and between LFIA and PCR (χ^2^ = 2.500, df = 1, *p* = 0.1138). These results indicate an agreement between the outcomes in both pairs.

The differences in TPR can be attributed to the fact that the sensitivity of immunoanalytical test systems is generally lower than that of real-time PCR. This means that samples with low levels of *P. brasiliense* may be incorrectly identified as negative using either ELISA or LFIA. The variations in FPR could suggest the presence of cross-reactivity with a closely related species of *Pectobacterium* that was not identified. Alternatively, this could be due to the absence of a PCR method as a reference, which may arise from genetic changes in the tested *P. brasiliense* isolates. Thus, test systems based on the obtained polyclonal antibodies can be used to detect infections caused by *P. brasiliense* bacteria. To our knowledge, the developed ELISA and LFIA are the first antibody-based assays for detection of *P. brasiliense*. Currently, no such test systems are mentioned in the literature or available in the catalogs of major manufacturers. This is likely because *P. brasiliense* was only recently identified as a separate species, and commercial kits are expected to become available in the near future. The test systems will be effective for rapid screening directly at the place of need (phytosanitary control sites, field monitoring, seed testing) or in laboratories with poor equipment.

## 4. Conclusions

*P. brasiliense* causes serious and destructive diseases in potatoes and other plants that lead to significant economic losses worldwide. A comprehensive approach that includes immunochemical detection methods is required for timely diagnosis and prevention of outbreaks. In this study, antibodies with high selectivity for *P. brasiliense* and differentiating it from closely related species *Pectobacterium* and *Dickeya* were obtained for the first time. Based on the obtained polyclonal antibodies, immunoassay test systems were developed: ELISA (detection limit equaled to 1.5 × 10^4^ cells/mL) and LFIA (detection limit equaled to 1 × 10^5^ cells/mL). The systems specifically recognize *P. brasiliense*. The sensitivity of the LFIA is inferior to the ELISA, but the analysis time (15 min) and the simplicity of its implementation are appropriate for rapid out-of-laboratory analysis of potato material. The application of the developed techniques for tuber material with *P. brasiliense* bacteria showed a high correlation between the ELISA and LFIA data. Therefore, the results show for the first time that polyclonal antibodies are feasible for application in ELISA and LFIA to detect *P. brasiliense*. Furthermore, these proposed methods of monitoring *P. brasiliense* are cost-effective and can be used under field conditions.

## Figures and Tables

**Table 1:** Results of testing bacterial cultures (10^8^ cells/mL) and potato tuber extracts with a double-antibody sandwich ELISA.

No.	Bacterium	Strain	ELISA	
			OD_450_	Assay Result
1	*Pectobacterium brasiliense*	F126	1.23	+
2	*Pectobacterium atrosepticum*	DSM 18077	0.11	-
3	*Pectobacterium atrosepticum*	VNIIF 5128	0.07	-
4	*Pectobacterium atrosepticum*	VNIIKR 0143	0.10	-
5	*Pectobacterium wasabie*	DSM 18074	0.07	-
6	*Pectobacterium be* *tavasculorum*	DSM 18076	0.10	-
7	*Pectobacterium carotovorum*	VNIIF 3391	0.09	-
8	*Pectobacterium carotovorum*	VNIIF 3398	0.17	-
9	*Pectobacterium* *odoriferum*	DSM 22566	0.07	-
10	*Pectobacterium carotovorum*	VNIIF C966	0.08	-
11	*Pectobacterium carotovorum*	DSM 30168	0.07	-
12	*Clavibacter sepedonicus*	VNIIKR 0435	0.11	-
13	*Clavibacter sepedonicus*	VNIIKR 0668	0.07	-
14	*Clavibacter michiganensis*	VNIIKR 0496	0.06	-
15	*Dickeya solani*	DSM 28711	0.09	-
16	*Dickeya dadantii* subsp. *dadantii*	DSM18020	0.07	-
17	*Dickeya paradisiaca*	DSM 18069	0.09	-
18	*Dickeya fangzhongdai*	DSM 101947	0.08	-
19	*Dickeya dadantii* subsp. *dieffenbachiae*	DSM 18012	0.07	-
20	*Dickeya dianthicola*	CFBP 3705	0.11	-
21	*Dickeya zeae*	DSM 18068	0.07	-
22	*Dickeya dianthicola*	VNIIKR 0144	0.09	-
23	*Ralstonia solanacearum*, race 3, bv.2	NCPPB 2316	0.07	-
24	*Pseudomonas fluorescence*	VNIIKR 0213	0.08	-
-	*Tuber extract from healthy potato*		0.09	-
+	*Tuber extract from healthy potato with added P. brasiliense*		1.65	+

**Table 2:** Results of testing tuber material using ELISA, LFIA and real-time PCR for detection of *P. brasiliense*.

Sample No	Potato Variety	ELISA		LFIA		Real-Time PCR	
		OD_450_	Assay Result	Color Intensity of Test Zone	Assay Result	Ct	Assay Result
1	Lady Claire	0.45	+	0.00	-	38	+
2	VR 808	0.34	+	0.00	-	28	+
3	Opal	0.22	-	0.00	-	no	-
4	Papageno	0.11	-	0.00	-	no	-
5	Lady Claire	0.24	-	0.00	-	no	-
6	Austin	0.47	+	2.79	+	no	-
7	Riviera	0.22	-	0.00	-	no	-
8	Lady Claire	0.17	-	0.00	-	no	-
9	Red Scarlet	0.09	-	0.00	-	no	-
10	Vineta	0.15	-	0.00	-	no	-
11	Asterix	0.07	-	0.00	-	38	+
12	Gala	0.07	-	0.00	-	38	+
13	Prime	0.06	-	0.00	-	no	-
14	Unknown	0.06	-	0.00	-	no	-
15	Unknown	0.69	+	9.09	+	30	+
16	Unknown	0.08	-	0.00	-	38	+
17	Sudarynya	0.07	-	0.00	-	no	-
18	Sudarynya	0.09	-	0.00	-	32	+
19	Sudarynya	0.08	-	0.00	-	35	+
20	Sudarynya	1.12	+	20.25	+	31	+
21	Sudarynya	0.07	-	0.00	-	no	-
22	Belaroza	0.06	-	0.00	-	no	-
23	Belaroza	0.06	-	0.00	-	36	+
24	Belaroza	0.06	-	0.00	-	no	-
25	Belaroza	0.05	-	0.00	-	no	-
26	Belaroza	0.11	-	0.00	-	no	-
27	Lady Claire	1.03	+	12.53	+	31	+
28	VR 808	1.14	+	19.05	+	32	+
29	Austin	1.10	+	23.15	+	33	+
30	Lady Claire	1.16	+	22.23	+	33	+
31	Prado	1.12	+	18.53	+	32	+
32	VR 808	0.62	+	19.01	+	24	+
33	Austin	0.74	+	11.47	+	34	+
34	Papageno	0.61	+	0.53	+	36	+
35	Lady Claire	0.50	+	0.00	-	no	-
36	Fontane	0.70	+	2.54	+	36	+
37	Opal	1.00	+	16.22	+	31	+
38	Lady Claire	0.91	+	20.56	+	33	+
39	Opal	1.13	+	20.99	+	33	+
40	Gala	0.39	+	1.40	+	no	-
41	Prime	0.71	+	19.33	+	24	+
42	Carmen	0.80	+	17.52	+	23	+
43	Unknown	0.10	-	0.00	-	no	-

## Data Availability

The raw data supporting the conclusions of this article will be made available by the authors on request.

## Supplementary Materials

The following supporting information can be downloaded at: https://www.mdpi.com/article/10.3390/microorganisms12122436/s1.
