# Supplementary material for: Highly Targeted Detection of Priority Phytopathogen Pectobacterium brasiliense: From Obtaining Polyclonal Antibodies to Development and Approbation of Enzyme-Linked Immunoassay and Lateral Flow Immunoassay
Source: Microorganisms. 2024 Nov 27;12(12):2436. doi: 10.3390/microorganisms12122436 (PMC11676143; doi:10.3390/microorganisms12122436)
Supplement: Supplementary file 1 [file microorganisms-12-02436-s001.zip › SI-revised.pdf]

Supporting information

Highly Targeted Detection of Priority Phytopathogen *Pectobacterium brasiliense*: From Obtaining Polyclonal Antibodies to Development and Approbation of Enzyme-Linked Immunoassay and Lateral Flow Immunoassay

Irina V. Safenkova [1], Pavel A. Galushka [2], Yuri A. Varitsev [2], Maria V. Kamionskaya [1], Natalia V. Drenova [3], Anna A. Vasilyeva [4], Anatoly V. Zherdev [1], Alexander I. Uskov [2] and Boris B. Dzantiev [1,*]

[1] A.N. Bach Institute of Biochemistry, Research Centre of Biotechnology of the Russian Academy of Sciences, 119071 Moscow, Russia; safenkova@inbi.ras.ru (I.V.S.); mari-kam@mail.ru (M.V.K.); zherdev@inbi.ras.ru (A.V.Z.)
[2] Russian Potato Research Centre, 140051 Kraskovo, Moscow region, Russia; pavel_galushka@mail.ru (P.A.G.); varyuriy@yandex.ru (Y.A.V.); korenevo2005@mail.ru (A.I.U.)
[3] All-Russian Plant Quarantine Centre, 140150 Bykovo, Moscow region, Russia; drenova@mail.ru
[4] Department of Plant Protection, Russian State Agrarian University—Moscow Timiryazev Agricultural Academy, 127434 Moscow, Russia; annadacyk@rgau-msha.ru
* Correspondence: dzantiev@inbi.ras.ru; Tel.: +7-495-954-3142

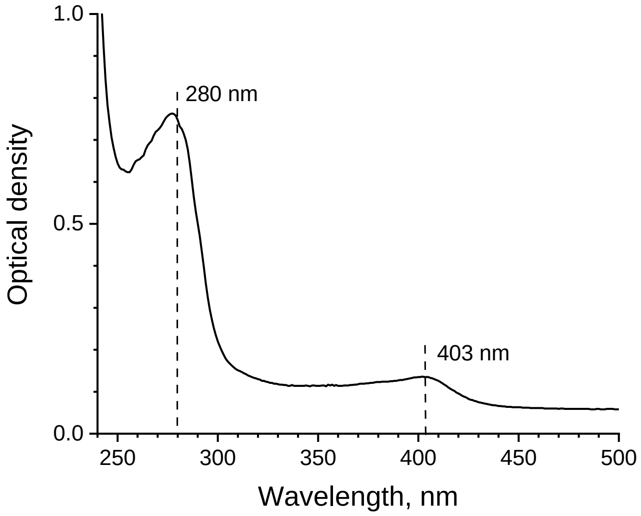

Figure S1. Absorption spectrum of the conjugate of antibodies with horseradish peroxidase. The maximum at a wavelength of 403 nm corresponds to the absorption of the peroxidase heme, and the maximum at a wavelength of 280 nm corresponds to the absorption of the antibody and peroxidase.

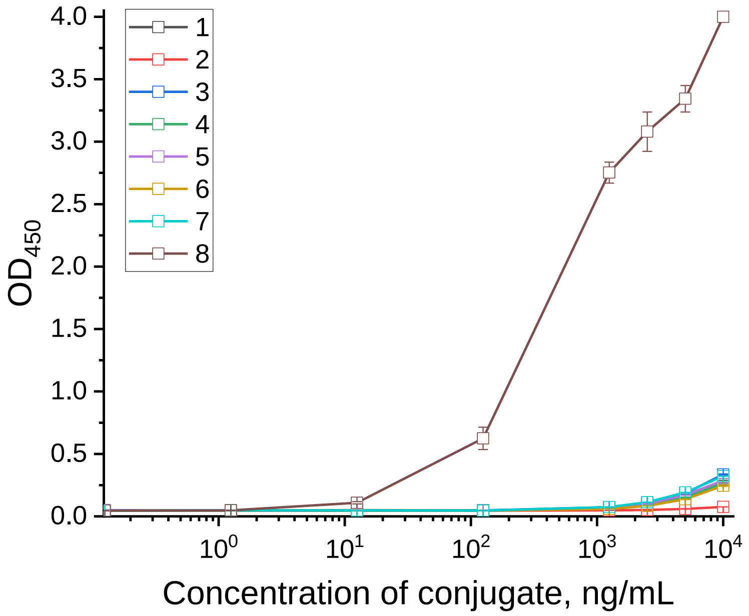

Figure S2. Characteristics of the conjugate of antibodies with horseradish peroxidase using the direct ELISA method with sorption of different bacteria in microplate wells at a concentration of 1 x 10^8 cells/mL: 1 – *D. solani*, 2 – *C. sepedonicus*, 3 – *E. amylovora*, 4 – *P. carotovorum,* 5 – *D. dianthicola*, 6 – *R. solanacearum*, *7 – P. atrosepticum,* 8 – *P. brasiliense*. Points at the curves are the average values of four replicates, error bars represent standard deviations.

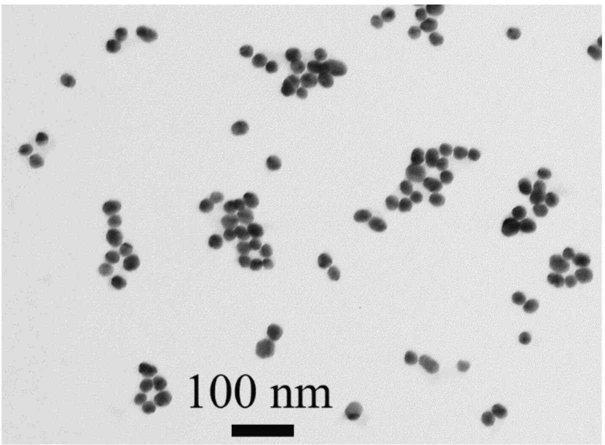

(A)

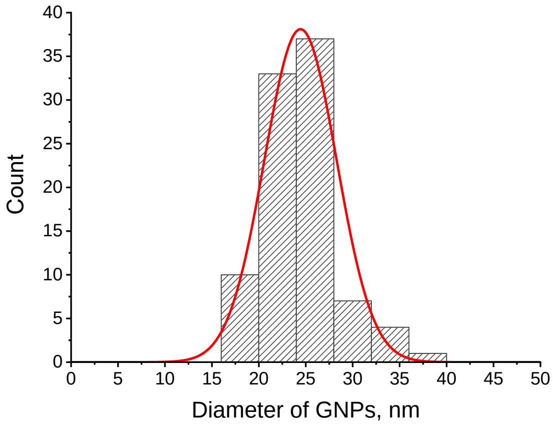

(B)

Figure S3. Micrograph of GNPs (A) and diameter distribution (number of particles = 92) of GNPs (B) by TEM.

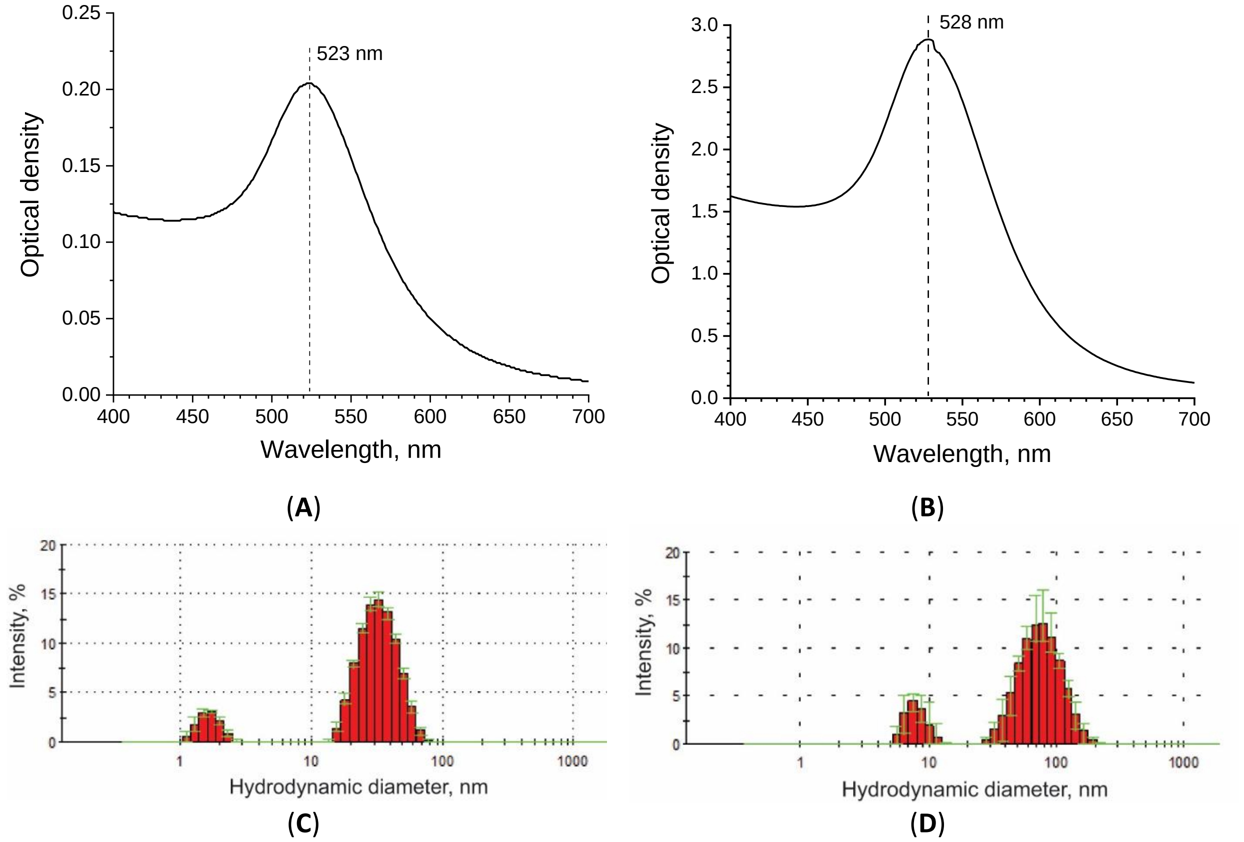

Figure S4. Characterization of the synthesized GNPs and their conjugates with pAbs. Absorption spectra of GNPs (**A**) and GNP-pAb conjugate (**B**); hydrodynamic diameter distributions obtained by DLS for GNPs (**C**) and GNP-pAb conjugate (**D**);

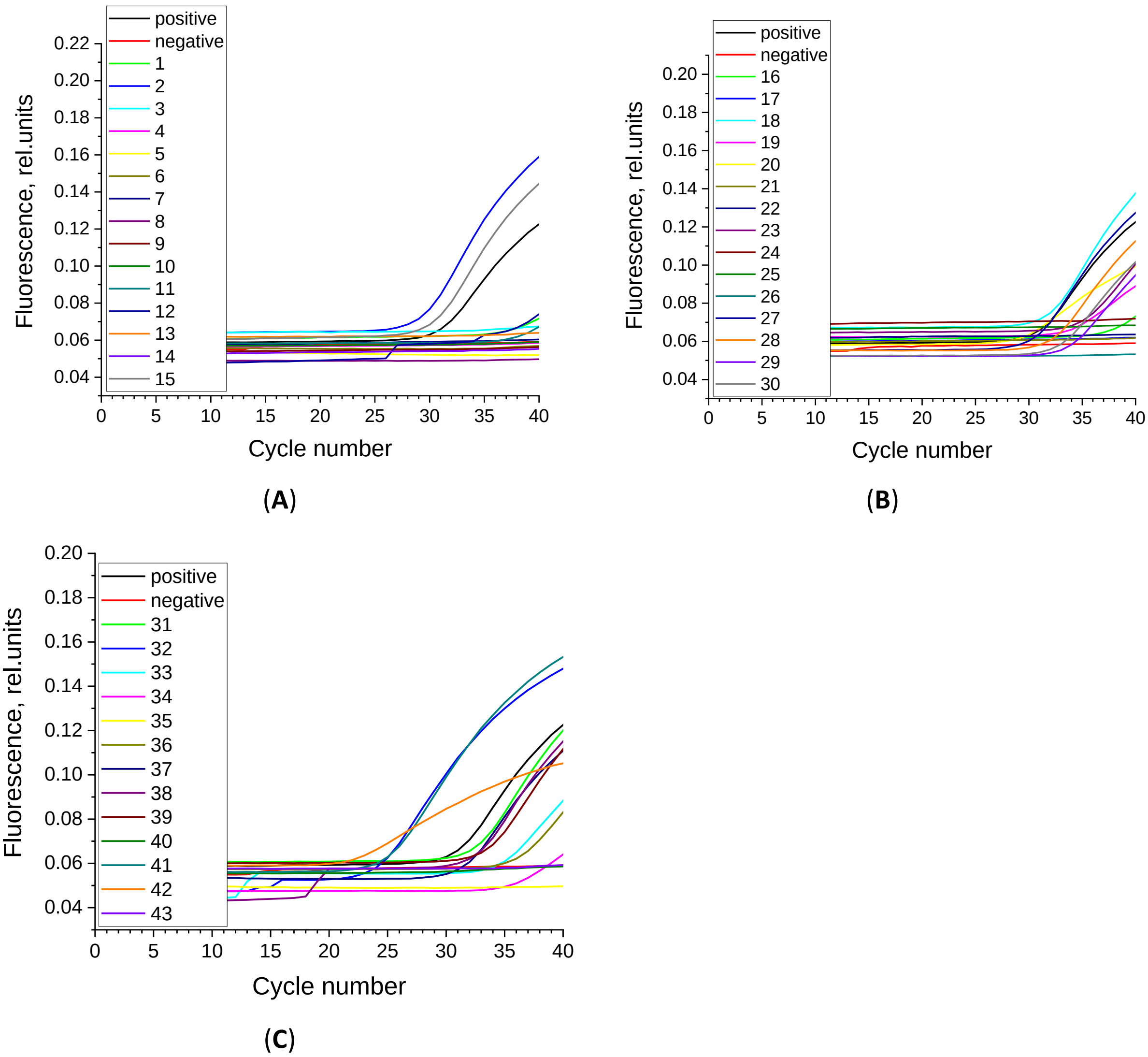

Figure S5. Fluorescence curves obtained using real-time PCR for testing tuber material for contamination with *P. brasiliense*: All samples are listed in Table 1: (**A**) 1-15, (**B**) 16-30, (**C**) 31-43 samples.

Table S1. Results of testing bacterial cultures (10^8 cells/mL) and potato tuber extracts with LFIA

**Bacterium**	**Test zone** **Control zone**
Dickeya solani	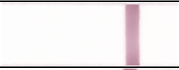
Clavibacter sepedonicus	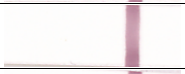
Erwinia amylovora	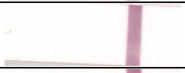
Pectobacterium carotovorum	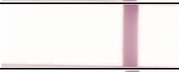
Dickeya dianthicola	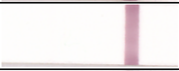
Ralstonia solanacearum	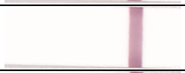
Pectobacterium atrosepticum	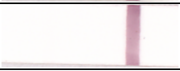
